# HBsAg Inhibits IFN-α Production in Plasmacytoid Dendritic Cells through TNF-α and IL-10 Induction in Monocytes

**DOI:** 10.1371/journal.pone.0044900

**Published:** 2012-09-14

**Authors:** Bisheng Shi, Guangxu Ren, Yunwen Hu, Sen Wang, Zhanqing Zhang, Zhenghong Yuan

**Affiliations:** 1 Shanghai Public Health Clinical Center, Fudan University, Shanghai, People’s Republic of China; 2 Laboratory of Molecular Virology, Shanghai Medical College, Fudan University, Shanghai, People’s Republic of China; Kantonal Hospital St. Gallen, Switzerland

## Abstract

Type I Interferon (IFN) is one of the first lines of defense against viral infection. Plasmacytoid dendritic cells (pDCs) are professional IFN-α-producing cells that play an important role in the antiviral immune response. Previous studies have reported that IFN-α production is impaired in chronic hepatitis B (CHB) patients. However, the mechanisms underlying the impairment in IFN-α production are not fully understood. Here, we report that plasma-derived hepatitis B surface antigen (HBsAg) and HBsAg expressed in CHO cells can significantly inhibit toll like receptor (TLR) 9-mediated Interferon-α (IFN-α) production in peripheral blood mononuclear cells (PBMCs) from healthy donors. Further analysis indicated that monocytes participate in the inhibitory effect of HBsAg on pDCs through the secretion of TNF-α and IL-10. Furthermore, TLR9 expression on pDCs was down-regulated by TNF-α, IL-10 and HBsAg treatment. This down-regulation may partially explain the inhibition of IFN-α production in pDCs. In conclusion, we determined that HBsAg inhibited the production of IFN-α by pDCs through the induction of monocytes that secreted TNF-α and IL-10 and through the down-regulation of TLR9 expression on pDCs. These data may aid in the development of effective antiviral treatments and lead to the immune control of the viral infections.

## Introduction

More than 350 million people worldwide are chronically infected with hepatitis B virus (HBV), and chronic infection with HBV causes significant morbidity and mortality [1],[2]. It is estimated that most neonates and approximately 5% of adults progress to chronic HBV infection following acute infection. Antiviral therapy can suppress HBV, but it cannot eliminate HBV infection entirely. Additionally, interferon-α (IFN-α), which is one of the most commonly used treatment for HBV infection, is only effective in half of all chronic HBV-infected patients who received the IFN-α treatment. Accumulating evidence indicates that an inadequate immune response to HBV is responsible for viral persistence [3], [4].

Plasmacytoid dendritic cells (pDCs) contribute to at least 95% of all of the IFN-α production among peripheral blood mononuclear cells (PBMCs). pDCs constitutively express the pattern recognition receptors toll like receptor (TLR) 7 and TLR9 [5], [6], [7]. TLR ligand stimulation or the detection of pathogen associated molecular patterns (PAMPs) can activate the myeloid differentiation factor 88 (MyD88)-interferon regulatory factor 7 (IRF-7) signaling pathway and result in high amounts of IFN-α secretion by pDCs [8]. Previous reports have indicated that both a reduced frequency and an impaired function of pDCs are observed in CHB patients. Following lamivudine treatment, a reduced HBV DNA level was observed to accompany an increased pDCs amounts. This suggested that HBV is important in the observed deficiency in the function of pDCs [9], [10]. However, the mechanism underlying the impairment of pDC function has not been fully elucidated.

It has been reported that HBV and the HBV surface antigen can enter dendritic cells, but the cells do not support viral replication [11]. This suggests a possible role of the HBV surface antigen in the impairment of pDC function. HBV surface antigen (HBsAg) is the subviral form of HBV envelope protein, and it contains multiple lipid modifications from the host hepatocyte and o-glycosylation [12], [13]. HBsAg consists of a small (S), a middle (M) and a large (L) protein that are all encoded by the same open reading frame (ORF), but translation is initiated at three distinct start codons. It has been reported that HBsAg is found in excess concentrations relative to the concentration of the HBV virion in CHB patients [14]. In some CHB patients, HBsAg concentrations have been reported to be as high as 100 µg/ml. However, there are still conflicting reports regarding the role of HBV and HBsAg in the inhibition of pDC function. Our previous study demonstrated that HBsAg could inhibit the IFN-α production of pDCs through the binding of HBsAg to the inhibitory receptor BDCA-2, thus leading to the down-regulation of IRF-7 nuclear translocation [15]. Woltman and colleagues have reported that HBV and HBsAg could abrogate the CpG-A/TLR9-induced mammalian target of rapamycin (mTOR)-mediated S6 phosphorylation, leading to IRF7 phosphorylation and IFN-α gene transcription. Additionally, HBV/HBsAg was also shown to inhibit the upregulation of co-stimulatory molecules, the production of TNF-α, IP-10 and IL-6 and pDC-induced NK cell function [16]. However, Vincent, I.E et al. have reported that HBV, but not HBsAg, can inhibit IFN-α production and TLR9 expression in pDCs [17]. HBsAg has also been reported to inhibit the TLR4-mediated cytokine production and CD80 upregulation in mDCs [18].

Herein, we further demonstrate that HBsAg can inhibit the production of IFN-α by pDCs in an indirect manner. HBsAg induced the secretion of TNF-α and IL-10 in monocytes, and these cytokines down-regulated the expression of TLR9 in pDCs to subsequently inhibit IFN-α production by pDCs.

## Materials and Methods

### Blood Donors

Five milliliters of EDTA-treated anti-coagulated peripheral blood specimens were collected from each chronic hepatitis B (CHB) patient at the Shanghai Public Health Clinical Center (SHAPHC) of Fudan University. CHB patients that were co-infected with HIV, HCV, HDV or HAV were excluded from this study. Patients PBMCs were isolated on recipient and stored in liquid nitrogen for further analysis. Plasma samples were stored at −80°C until required. Buffy coat from healthy donors were collected from the Shanghai (Red Cross) Blood Center.

### Ethics Statement

This study was reviewed and approved by the Ethics Committee of SHAPHC and all participants were given written informed consent before inclusion in the study.

### Cells and Reagents

Human pDCs were isolated with a BDCA-4-positive isolation kit (Miltenyi Biotec) and monocytes were isolated with a CD14-positive selection kit (Stem cell). Recombinant human IL-3 (PeproTech) at a concentration of 10 µg/ml was added to the pDCs culture to sustain its survival. To stimulate the pDCs, 1 µg/ml GardiQuimod (Invivogen) and 3 µM CpG ODN2216 (purchased from Invivogen or synthesized from Invitrogen Shanghai) were used. Recombinant human IL-10 and recombinant human TNF-α were purchased from PeproTech and R&D Systems, respectively. LEAF™-purified anti-human TNF-α and IL-10 neutralization antibodies were both purchased from Biolegend. The neutralization antibody isotype controls were purchased from eBioscience. BDCA-2-APC was purchased from Miltenyi Biotec and CD123-FITC was purchased from Biolegend. The IFN-α-PE that was used for the intracellular staining assay was purchased from BD Bioscience. TLR9-PE was purchased from eBioscience.

### Characterization of the HBV Surface Antigen

Plasma-derived HBsAg (pHBsAg) purified from the serum of HBV patients (kindly provided by Kehua Bio-engineering Co. Ltd., Shanghai, China) was described previously [15]. Briefly, the serum from CHB patients was inactivated with formaldehyde followed by sucrose density gradient centrifugation and affinity chromatography to purify the antigens and was subsequently diluted in phosphate buffered saline (PBS). The pHBsAg was used to immunize animals, and the antibody that was generated against HBsAg was used to prepare the ELISA kit. HBsAg produced in Chinese hamster ovary (CHO) cells (CHO-HBsAg) was purchased from the NCPC GeneTech Biotechnology development Co. Ltd. No adjuvant was added to the CHO-HBsAg. Both the patient-derived and the CHO cell-derived HBsAg samples were run on a 12% SDS-PAGE for subsequent silver staining and western blotting. For the western blot analysis, horseradish peroxidase-labeled HBsAb (HRP-HBsAb) (Kehua Bio-engineering Co. Ltd.) was used to detect the 24 kD HBsAg and the glycol forms of the protein. The HBsAg was also analyzed by transmission electron microscopy (TEM) to determine the particle size and morphology, as described in our previous studies [15].

### Preparation of Plasmacytoid Dendritic Cells and Monocytes

PBMCs were obtained by Ficoll-Hypaque (Lymphoprep™, Axis-Shield, Oslo, Norway) gradient centrifugation following the manufacturer’s instructions. The PBMCs were cultured at 2×10^6/^ml in 96-well plates in RPMI 1640 medium (Gibco) with 10% FBS (Gibco), 100 U/ml penicillin, 0.1 g/L streptomycin and 1% nonessential amino acids at 37°C with humidified air containing 5% CO_2_. pDCs were isolated from the freshly prepared PBMCs by positive selection with anti-BDCA-4 antibody-coated magnetic beads, according to the manufacturer’s instructions (Miltenyi Biotec). Briefly, 1×10^8^ PBMCs were resuspended in a volume of 300 µl of separation buffer (ice-cold PBS, 0.5% BSA and 2 mM EDTA) and incubated with 100 µl FcR-blocking reagent and 100 µl anti-BDCA-4 microbeads for 15 minutes at 4°C. Then, the PBMCs were washed with 20 times the volume of the cell mixture with cold separation buffer (10 ml/10^8^ cells) and selected in a magnetic column. The magnetic separation was repeated using a new column to increase purity. The purity of the pDCs was determined by staining with BDCA-2-FITC and CD123-PE pDC markers and analyzing the cells through flow cytometry. The purity of the pDCs was >95%. For the co-culture and IFN-α production assays, pDCs were cultured in RPMI 1640 medium with 10% FBS and 10 µg/ml recombinant human IL-3 in the absence or presence of TLR9 ligands.

Monocytes were separated from the PBMCs using anti-CD14 coated magnetic beads (Stem cell), according to the manufacturer’s instructions. Briefly, PBMCs (1×10^8^ cells/ml) were incubated with the Easysep CD14 positive selection cocktail (100 µl/ml) for 15 minutes at room temperature. Then, Easysep magnetic nanoparticles (50 µl/ml) were added to the cell suspension for another 10 minutes to bind to the positive cells. After incubation, the total volume of the cell suspension was adjusted to 2.5 ml by adding PBS supplemented with 0.5% BSA and 1 mM EDTA. Subsequently, the cell suspension was placed in a magnetic field for 5 minutes to capture the magnetic nanoparticles bound to the monocytes. The cells in the magnetic field were carefully washed twice to increase the purity. The purity of the monocytes was determined by staining with a PE-anti-human CD14 antibody and analyzing the cells by flow cytometry. The purity of the monocytes was consistently >95%. The density of the monocytes was adjusted, and the cells were cultivated in RPMI 1640 medium.

### Intracellular Cytokine Staining and Flow Cytometry Assay

Intracellular cytokine staining (ICS) and flow cytometry were used to measure the IFN-α producing ability of PBMCs from CHB patients and healthy donors and the TLR9 expression of pDCs. Briefly, PBMCs were seeded (1×10^6^ cells/well) in 96-well culture plates and treated with TLR9 ligands (3 µM CpG ODN2216) for 2 hours at 37°C. Brefeldin A (1∶1000) was added to the cell culture for another 3 hours. The PBMCs were then collected and washed twice with washing buffer (PBS supplemented with 2% FBS) and resuspended in a final volume of 45 µl. The PBMCs were then stained with antibodies against BDCA-2 (APC-labeled, Miltenyi Biotec) and CD123 (FITC-labeled, Biolegend) at 4°C for 30 minutes in dark to allow the identification of the pDC population. Subsequently, the cells were washed once, resuspended in PBS and fixed at room temperature for 20 minutes. The cells were then washed twice with permeabilization buffer and resuspended in permeabilization buffer for 1 hour. The cells were then washed twice with permeabilization buffer and resuspended in 45 µl buffer. PE-anti-human IFN-α2b (5 µl, BD Biosciences) was added, and the cells were incubated at 4°C for 30 minutes in the dark. The cells were washed with permeabilization buffer twice and resuspended in 200 µl buffer. The FACS analysis was performed using a FACSAria (BD Biosciences). The data were analyzed using the Flowjo software (TreeStar.San Marcos, CA). TLR9 expression was also stained as described above and the mean fluorescent intensity (MFI) shift was shown.

### Transwell Cultures

A Transwell culture assay was utilized to investigate the interaction between the pDCs and monocytes. pDCs were seeded in the upper compartment of a 24-well Transwell chamber (0.3 µm pore size membrane) at a cell density of 4×10^4^ cells/well. Monocytes were cultured in the lower chamber at a cell density of 8×10^5^ cells/well. The pDCs and monocytes were co-cultured in the Transwell chambers for 2 hours at 37°C in the presence of IL-3 (10 µg/ml). The cells were then pretreated with or without HBsAg (2 µg/ml) for 20 hours, after which they were stimulated with the TLR9 ligand CpG ODN2216 (3 µM) for an additional 24 hours. The cell supernatants were collected and measured using the Human IFN-α ELISA Kit (PBL Biomedical laboratories).

### Measurement of the Cytokine Levels

Plasma samples that were harvested from CHB patients and healthy donors were stored at −80°C until the measurement of cytokines was conducted. The cytometric bead array (CBA kit; BD Bioscience) was used to determine the levels of IL-1β, IL-6, IL-8, IL-10, IL-12 and TNF-α, according to the manufacturer’s instructions. Briefly, 50 µl of each plasma sample was mixed with 50 µl of pre-mixed capture beads and 50 µl of Human Inflammation PE Detection Reagent for 3 hours at room temperature in the dark. After incubation, the samples were washed once with washing buffer, resuspended in a final volume of 300 µl and analyzed by flow cytometry. The data were analyzed using the CBA software (BD Bioscience), and the concentration of each cytokine was determined by extrapolation against the corresponding standard curve.

For the IFN-α quantitation, the cell supernatants were collected and stored at −80°C until analysis. The cell supernatants were measured using the human IFN-α 1 ELISA kit (PBL Biomedical laboratories). For the detection of TNF-α and IL-10 in the cell culture supernatants, the human TNF-α ELISA kit and the human IL-10 ELISA kit (Invitrogen) were used according to the manufacturer’s instructions.

### Statistical Analysis

Student’s t test was performed using the Graphpad Prism software. All tests were two-tailed, and P-values <0.05 were considered significant. The symbol “n.s.” indicates that the value is non-significant, “*” indicates a P-value <0.05, and “**” indicates a P-value <0.01.

## Results

### 1. IFN-α Production is Impaired in PBMCs and pDCs from Treatment-naïve, Chronic Hepatitis B (CHB) Patients

**Figure 1:**
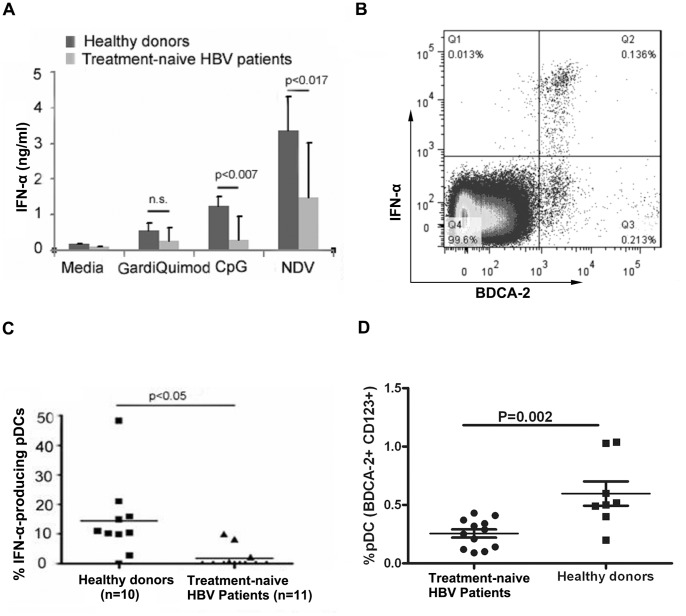
CHB patient-derived PBMCs had impaired TLR9-mediated IFN-α production. (A) Comparison of the ability of healthy donors and CHB patients to produce IFN-α. PBMCs (2×10^6^ cells) obtained from healthy donors (n = 20) and treatment-naïve CHB patients (n = 30) were stimulated with CpG ODN2216 (TLR9 ligand, 3 µM), GardiQuimod (TLR7 ligand 1 µg/ml) or NDV (3.12 HA units/ml) for 48 hours Cell culture supernatants were collected to measure IFN-α production. (B) PBMCs (2×10^6^ cells) from patients or healthy donors were stimulated with 3 µM CpG ODN2216 for 3 hours and brefeldin A were added to the culture supernatant while stimulation. The cells were then collected and stained with BDCA-2 and IFN-α. The IFN-α producing pDCs were then analysed. (C) Comparison of the frequency of pDCs in healthy donors and CHB patients. PBMCs from healthy donors (n = 10) and treatment-naïve CHB patients (n = 12) were surface stained with CD123-FITC and BDCA-2-APC to identify the pDC population by flow cytometry. The pDC frequency was then calculated for each healthy donor and patient. (D) Comparison of the functional pDCs ratio in healthy donors and CHB patients. PBMCs obtained from healthy donors (n = 10) and treatment-naïve CHB patients (n = 12) were stimulated with CpG ODN2216; then, the PBMCs were surface stained with CD123-PE and HLA-DR-PerCP to identify the pDC population. The cells were then fixed, and intracellular IFN-α staining was performed to identify the IFN-α producing cells. Dotted numbers indicate the percentage of IFN-α-positive cells.

IFN-α is critical for the innate immune control of HBV replication and for the activation of the adaptive immune response. pDCs account for most of the IFN-α production in PBMCs due to their high expression of TLR7, TLR9 and IRF-7. Given the importance of pDCs in controlling viral replication, there is speculation that the function of pDCs may be impaired by HBV during chronic HBV infection. Previous studies have demonstrated that the frequency of pDCs is decreased and the function of the pDCs is impaired in CHB patients. Therefore, we first confirmed these results. Thirty treatment-naïve, CHB patients and 20 healthy donors were enrolled in this study. PBMCs from each patient or healthy donor were isolated, and 4×10^5^ PBMCs were stimulated with the TLR9 ligand CpG ODN2216, the TLR7 ligand Gardiquimod or new castle virus (NDV) for 24 hours, as illustrated in Fig. 1A. The supernatants were then collected for IFN-α quantitation.

Data indicated that PBMCs from CHB patients had a significant reduction in IFN-α production in response to CpG ODN2216 and NDV stimulation when compared to the healthy donor PBMCs (Fig. 1A). However, the TLR7 signaling pathway in CHB patient PBMCs was not affected when compared to the healthy donor PBMCs (Fig. 1A). More importantly, the number of functional pDCs that produced IFN-α in response to CpG ODN2216 stimulation was also reduced in PBMCs of the CHB patients (Fig. 1B). Additionally, we compared the relative ratio of pDCs to PBMCs between the healthy donors and the CHB patients, as determined by FACS analysis, and confirmed that the frequency of pDCs was reduced (Fig. 1C).

### 2. HBsAg Inhibits the Production of IFN-α by PBMCs Isolated from Healthy Donors in vitro

**Figure 2:**
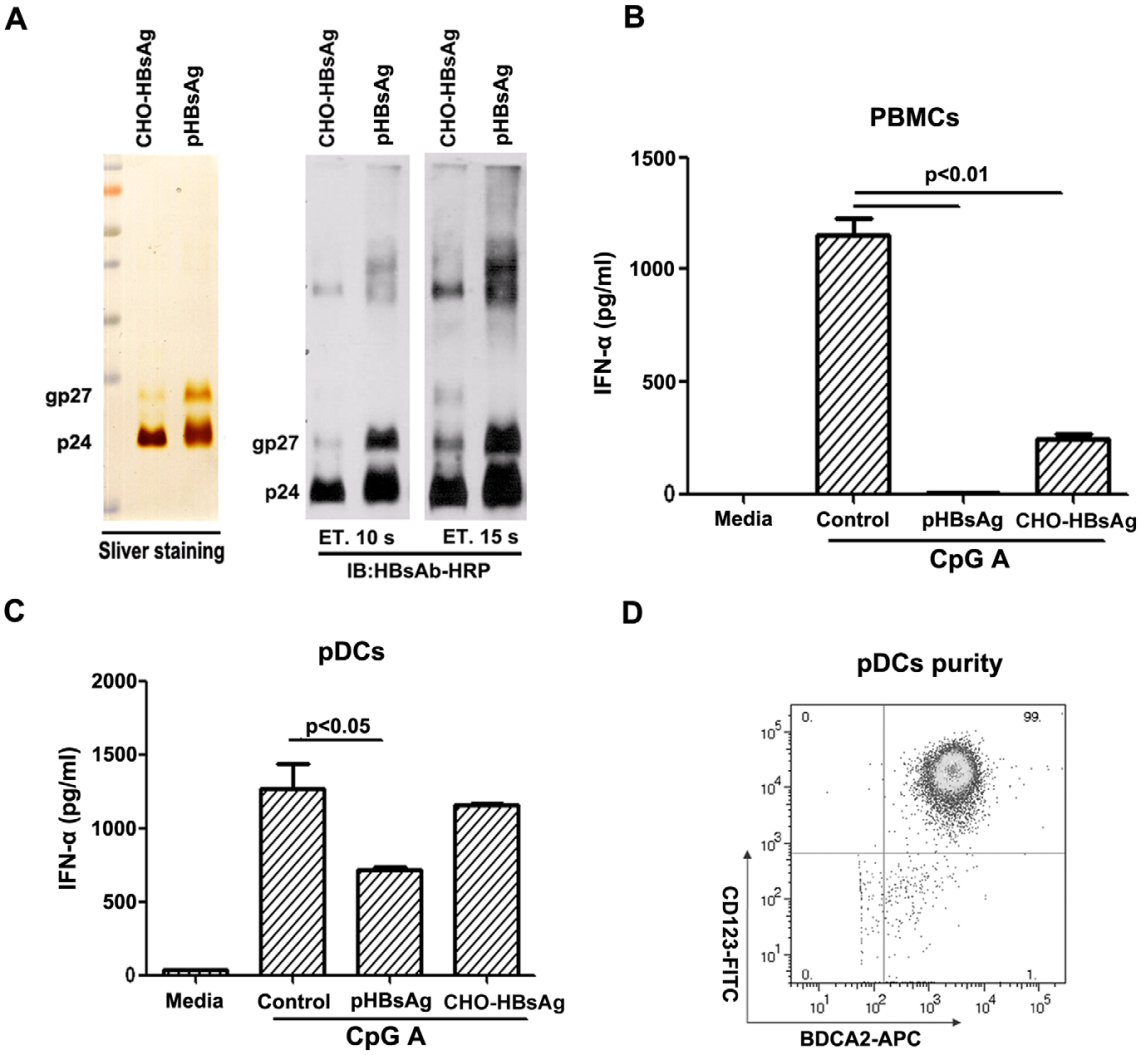
HBsAg inhibits the production of IFN-α in purified pDCs. (A) Characterization of the serum-derived HBsAg (pHBsAg) and HBsAg produced in CHO cells (CHO-HBsAg). 1 µg of pHBsAg and 0.76 µg of CHO-HBsAg were run on a 12% SDS-PAGE for subsequent silver staining and western blot analysis. The unglycosylated (24 kD) and glycosylated (27 kD) form of HBsAg were observed. (B) The inhibitory effects of pHBsAg and CHO-HBsAg on the production of IFN-α by PBMCs. PBMCs (2×10^6^/200 µl) from healthy donors were pretreated with pHBsAg (1 µg/ml) or CHO-HBsAg (2 µg/ml) for 24 hours followed by stimulation with the TLR9 ligand CpG ODN2216 (3 µM) for an additional 24 hours. The cell supernatants were collected for IFN-α quantitation. All of the assays were performed in duplicate, and these data are representative of at least five independent experiments. (C) The inhibitory effects of pHBsAg and CHO-HBsAg on the production of IFN-α by pDCs. pDCs from healthy donors were purified by magnetic bead isolation, cultured at a concentration of 1×10^4^/200 µl in 96-well cell culture plates and treated in the same manner as previously mentioned in 2B. The cell supernatants were collected for IFN-α quantitation. These data are representative of at least five independent experiments. (D) The purity of the pDCs was analyzed by FACS analysis.

It is well known that HBsAg is present at a 10,000 to 1,000,000 fold excess concentration compared to Dane particles. This indicates that HBsAg may have additional functions other than virion formation. We and others have determined that HBsAg is involved in the impairment of TLR9 ligand-stimulated IFN-α production in PBMCs. Here, we analyzed the inhibitory effect that different sources of HBsAg have on PBMCs. The quality of different HBsAg was first analyzed by sliver staining and western blotting. Serum-purified HBsAg (pHBsAg) was observed to have a typical two-band pattern that represents the unglycosylated 24 kD protein and the glycosylated 27 kD form, while the CHO cell-derived HBsAg (CHO-HBsAg) was observed to have less glycosylation as the 27 kD bands were not as distinct as those of the pHBsAg (Fig. 2A).

PBMCs isolated from healthy donors were incubated with pHBsAg or CHO-HBsAg for 24 hours followed by TLR9 ligand stimulation for additional 24 hours The cell culture supernatant was then collected, and the production of IFN-α was analyzed by ELISA. The results indicated that both pHBsAg and CHO-HBsAg significantly inhibited the secretion of IFN-α by PBMCs stimulated by TLR ligands, but pHBsAg showed a greater inhibitory effect than CHO-HBsAg (Fig. 2B).

Because pDCs are the main producers of IFN-α in PBMCs, we examined whether HBsAg could directly interfere with the function of pDCs or whether the inhibition occurred through an alternative pathway. pDCs were MACS isolated from PBMCs and the purity of the pDCs was >95% by FACS analysis (Fig. 2D). Purified pDCs were then incubated with pHBsAg or CHO-HBsAg, followed by stimulation with TLR9 ligands CpG ODN2216. The production of IFN-α was then assayed by ELISA. The results indicated that pHBsAg could inhibit the production of IFN-α in the purified pDCs, but the extent of inhibition was lesser than that observed in bulk PBMCs (Fig. 2C). These results suggested that in addition to the direct effect of HBsAg on pDCs, HBsAg may also inhibit IFN-α production in an indirect manner.

### 3. Monocytes Participate in the Inhibitory Effects of HBsAg on the Production of IFN-α by pDCs

**Figure 3:**
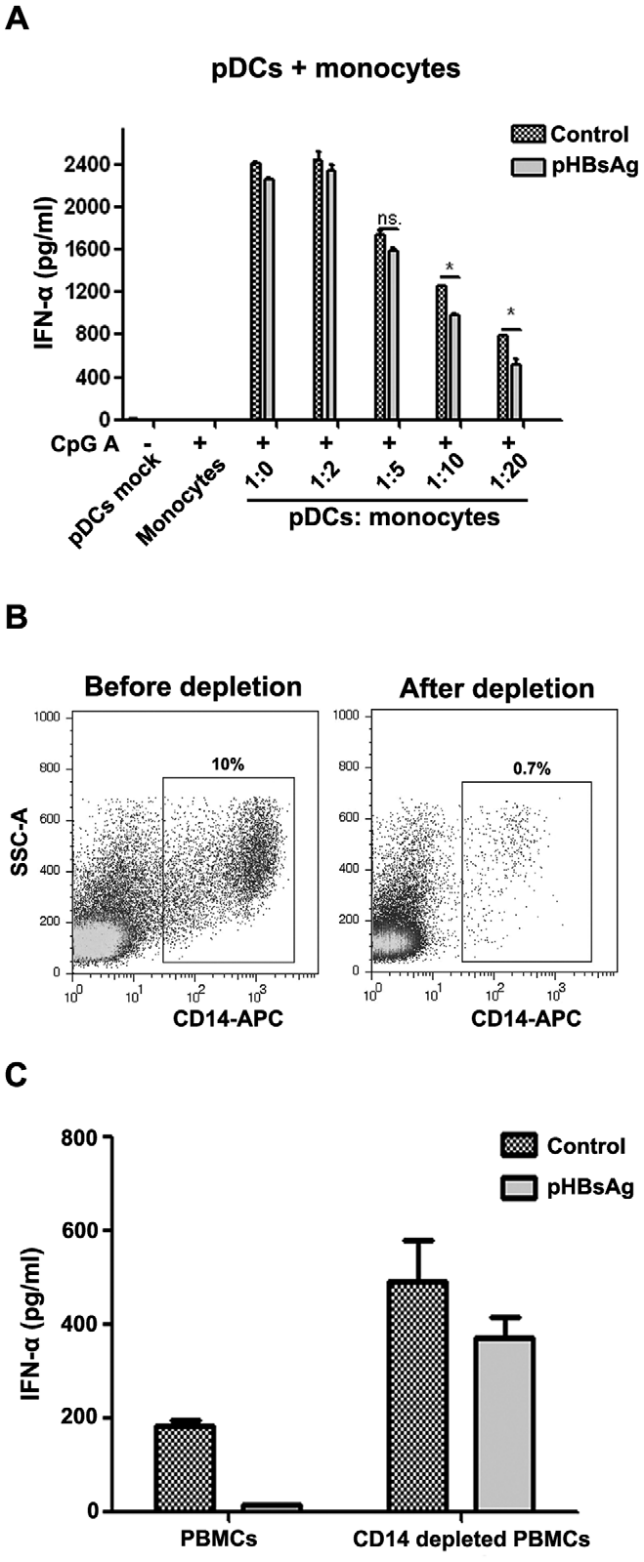
Monocytes participate in the inhibition of IFN-α production in PBMCs by HBsAg. (A) Magnetic bead-isolated monocytes and pDCs were co-cultured at varying ratios in RPMI 1640 medium supplemented with IL-3 (10 ng/ml) in 96-well plates. The pDCs (1×10^4^ per well) number was constant, and of the ratio of monocytes varied (0, 2, 5, 10, 20 fold). The cells were co-cultured and treated in the same manner as in 2B. The IFN-α production was determined by ELISA. The data are representative of at least three independent experiments. (B) The efficiency of CD14+ monocytes depletion. Monocytes were depleted using a CD14+ monocytes positive selection kit. The positive fraction was removed, and the negative fraction was collected. The purity of the depletion was determined by FACS analysis. (C) Total PBMCs and CD14+ monocyte-depleted PBMCs were treated in the same fashion as in 2B. IFN-α production was determined by ELISA. The data are representative of at least three independent experiments.

Monocytes are a major cell type in PBMCs and several previous reports have indicated that monocytes could negatively regulate pDCs functions during viral infection [19], therefore monocytes may participate in the inhibition of IFN-α production by pDCs. To confirm this hypothesis, monocytes were purified from PBMCs by magnetic bead isolation and were co-cultured with varying concentrations of pDCs. The co-culture was treated with 1 µg/ml HBsAg for 24 hours followed by stimulation with CpG ODN2216 for an additional 24 hours. The IFN-α secretion in the cell supernatant was then examined by ELISA. The results indicated that HBsAg did not inhibit the production of IFN-α when pDCs and monocytes were mixed at a ratio of 1∶1 or 1∶5. However, when the number of monocytes was 10 to 20-fold higher than the number of pDCs, the inhibitory effect of HBsAg on IFN-α secretion was significant (Fig. 3A). This increased ratio of monocytes to pDCs we used is similar to the physiological composition of PBMCs, where the ratio of monocytes to pDCs is nearly 20∶1.

To confirm the role of monocytes in the inhibitory effect of HBsAg, we depleted the CD14-positive monocytes in PBMCs using CD14 antibody-coated magnetic beads and determined whether HBsAg could still inhibit the production of IFN-α by PBMCs. The results indicated that the percentage of monocytes in PBMCs was reduced from 10% to 0.7% after monocytes depletion (Fig. 3B) and that the inhibitory effect of HBsAg on the production of IFN-α was nearly abolished after the monocytes were depleted from PBMCs, as compared to the intact PBMCs (Fig. 3C). Additionally, we examined if CD4-positive lymphocytes could exert a similar inhibitory effect, and these results indicated that the lymphocytes had no inhibitory effect on the production of IFN-α by pDCs (data not shown). These results indicated that monocytes play a key role in HBsAg’s inhibition of IFN-α production by pDCs.

### 4. HBsAg can Induce the Production of TNF-α and IL-10 by Monocytes

**Figure 4:**
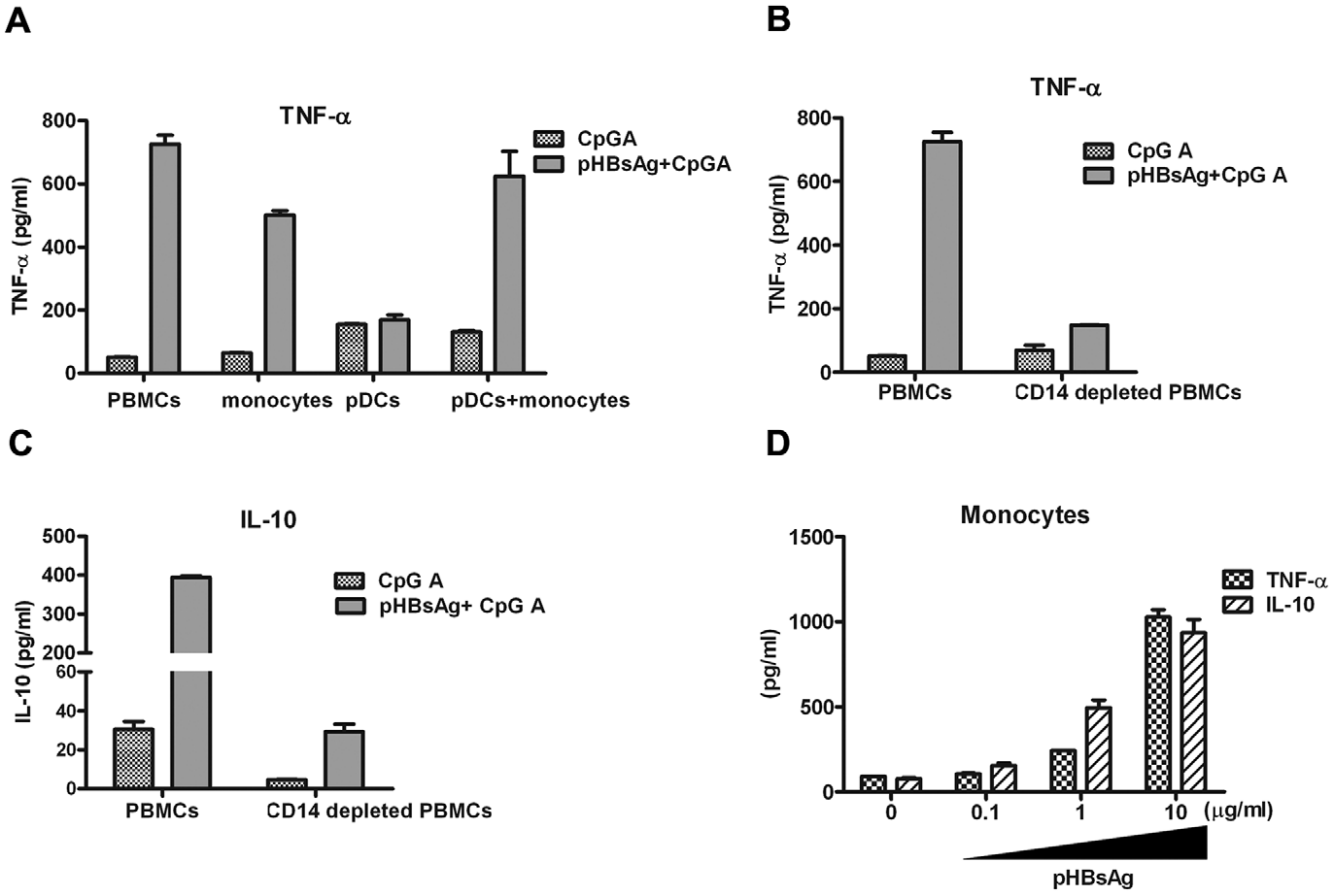
HBsAg induces the production of TNF-α and IL-10 in monocytes. (A) Monocytes and pDCs were isolated using magnetic beads. 3×10^4^ pDCs were either co-cultured or separated by a transwell insert with 6×10^5^ monocytes in a 24 well in 10% FCS-RPMI 1640 containing IL-3 (10 ng/ml). The cells were either pretreated or not with 1 µg/ml pHBsAg and stimulated with 3 µM CpG ODN2216 for additional 24 hours. The IFN-α production was determined by ELISA. The data shown are representative of at least three experiments. (B) 2×10^5^ monocytes and 1×10^4^ pDCs were co-cultured in 10% FCS-RPMI 1640 containing IL-3 (10 ng/ml) in 96-well culture plates in a total volume of 200 µl. The cells were pretreated with 1 µg/ml pHBsAg and stimulated with 3 µM CpG ODN2216 for 24 hours. The TNF-α production was determined by an ELISA. The data are representative of at least three experiments. (C) CD14+-depleted PBMCs were cultured at a concentration of 2×10^6^/200 µl in 96-well culture plates and then pretreated with 1 µg/ml pHBsAg followed by stimulation with 3 µM CpG ODN2216 for 24 hours. The TNF-α production was determined by an ELISA. The data are representative of at least three experiments. (D) PBMCs and CD14+-depleted PBMCs were cultured at a concentration of 2×10^6^/200 µl in 96-well culture plates and pretreated with 1 µg/ml pHBsAg followed by stimulation with 3 µM CpG ODN2216 for 24 hours. The IL-10 production was determined by an ELISA. The data are representative of at least three experiments. (E) Monocytes were isolated by magnetic bead treatment and cultured in 96-well plates at a concentration of 1×10^5^/200 µl. pHBsAg at a concentration of 1 µg/ml was added to the culture supernatant for 24 hours, and the production of TNF-α and IL-10 was determined by an ELISA.

To further study the mechanism of the monocyte-mediated HBsAg inhibition of IFN-α production by pDCs, a transwell assay was utilized to determine whether monocytes inhibited pDCs by a direct cell-to-cell interaction or by an indirect fashion, for example, by the secretion of cytokines. pDCs (4×10^4^/well) and monocytes (8×10^5^/well) were co-cultured or separated in a 0.3-µm pore transwell. Both cultures were treated with HBsAg, followed by stimulation with CpG ODN2216. The results indicated that HBsAg treatment resulted in a significant inhibition of IFN-α production in both the co-culture and the transwell culture (Fig. 4A). This indicated that the monocytes effect may be a result of the secretion of cytokines that can cross the membrane of the transwell.

Previous studies have reported that TNF-α and IL-10 can decrease the production of IFN-α by pDCs [20], [21]. Therefore, we examined whether HBsAg treatment could induce monocytes’ secretion of these cytokines. TNF-α and IL-10 levels were quantified in bulk PBMCs, purified monocytes, purified pDCs and co-cultured pDC-monocytes. The results indicated that after HBsAg treatment, TNF-α level was significantly elevated in bulk PBMCs, purified monocytes and co-cultured pDCs-monocytes but not in the purified pDCs (Fig. 4B). This indicated that the TNF-α secretion induced by HBsAg was mainly produced by monocytes. Additionally, TNF-α and IL-10 secretion was detected in the monocyte-depleted PBMC culture after treatment with HBsAg. PBMCs that were depleted of monocytes produced much less TNF-α and IL-10 compared to bulk PBMCs in response to HBsAg treatment (Fig. 4C and 4D). This suggested that the TNF-α and IL-10 induced by HBsAg treatment were secreted by monocytes, although small amounts of TNF-α and IL-10 was still induced in the monocyte-depleted PBMCs.

To further confirm the effect of HBsAg on monocytes, the production of cytokines by purified monocytes in response to varying concentrations of pHBsAg was analyzed. The results indicated that pHBsAg induced the production of TNF-α and IL-10 in a dose-dependent manner (Fig. 4E). To further confirm the effect of pHBsAg, CHO-HBsAg was used to stimulate the monocytes. As expected, CHO-HBsAg was also able to induce robust secretion of TNF-α and IL-10 by monocytes (data not shown).

### 5. HBsAg-induced TNF-α and IL-10 Secretion Mediates the Inhibition of IFN-α Production

**Figure 5:**
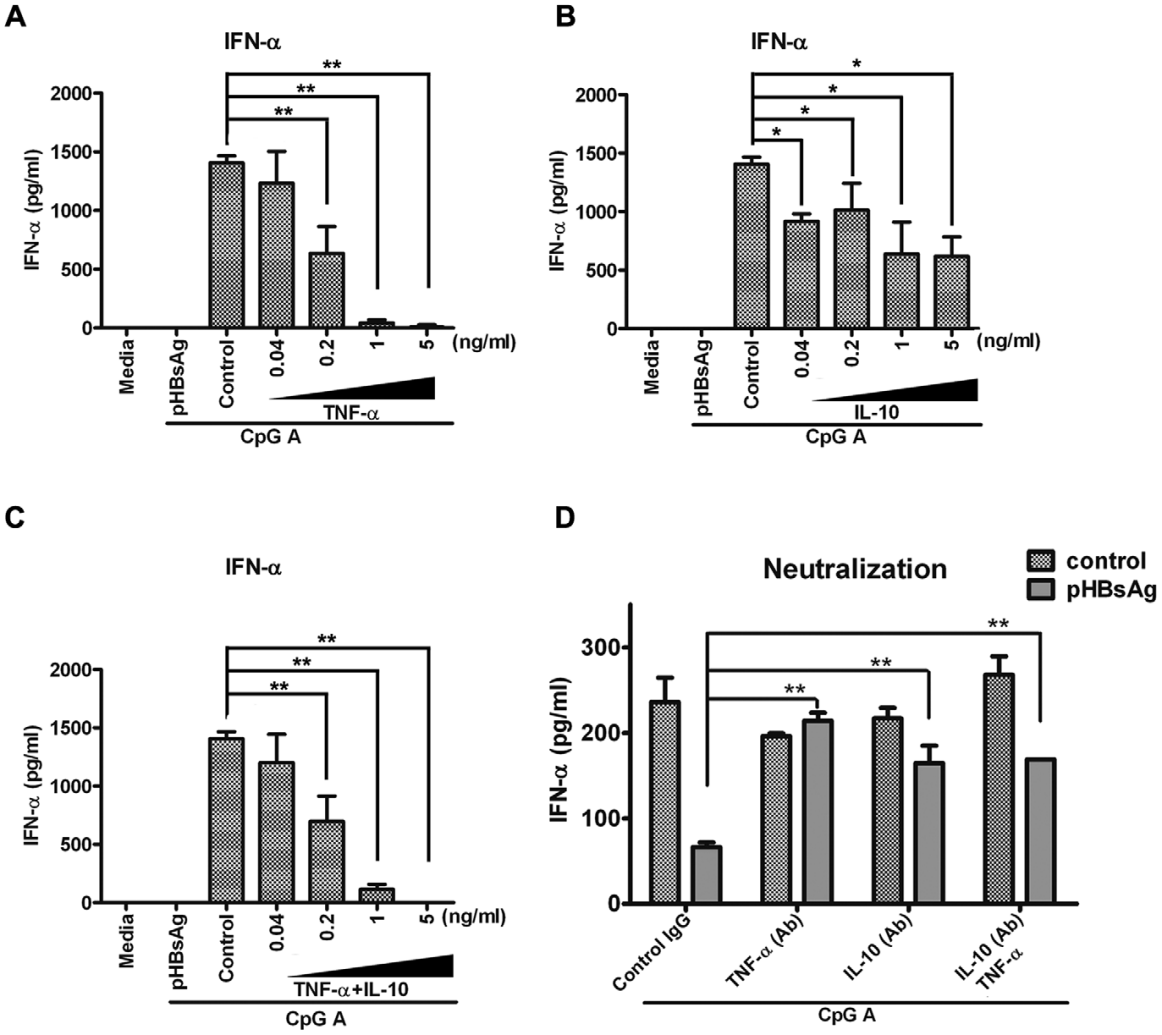
TNF-α and IL-10 induction by HBsAg may be responsible for IFN-α inhibition in PBMCs. (A, B, C) PBMCs at a concentration of 2×10^6^/200 µl were cultured in 96-well culture plates, and 10 ng/ml of exogenous TNF-α (A), IL-10 (B) or TNF-α and IL-10 together (C) was added to the PBMCs for 24 hours followed by stimulation with 3 µM CpG ODN2216 for 24 hours. IFN-α secretion was determined by an ELISA. The data are representative of at least three experiments. (D) PBMCs at a concentration of 2×10^6^/200 µl were cultured in 96-well culture plates, and 10 µg/ml TNF-α and IL-10 neutralization antibodies were added together to the PBMCs with 1 µg/ml HBsAg for 24 hours. The PBMCs were then stimulated with 3 µM CpG ODN2216 for 24 hours. The IFN-α expression level was quantified by an ELISA. Mouse normal IgG_3_ was used as a control. The data are representative of at least three experiments.

Because HBsAg induced the secretion of TNF-α and IL-10, we verified the inhibitory effects of these cytokines on the production of IFN-α by PBMCs. PBMCs were pretreated with different doses of both TNF-α and IL-10 for 24 hours followed by CpG ODN2216 stimulation for additional 24 hours IFN-α production was then assayed by ELISA. Both TNF-α and IL-10 inhibited the production of IFN-α in a dose-dependent manner (Fig. 5A and 5B). Additionally, the combination of TNF-α and IL-10 treatment also inhibited the production of IFN-α (Fig. 5C). Furthermore, the inhibitory effect of HBsAg treatment was reduced when neutralizing antibodies against these two cytokines were added during HBsAg treatment (Fig. 5D). These data support the hypothesis that TNF-α and IL-10 are involved in the inhibitory effect of HBsAg on IFN-α production. It should be noted that the PBMCs used in Fig. 5D are from different donors than those used in Fig. 5A, B, C. Therefore, the IFN-α production may vary with respect to the donor.

### 6. HBsAg Inhibits the Production of IFN-α by pDCs through the Down-regulation of TLR9 Expression

**Figure 6:**
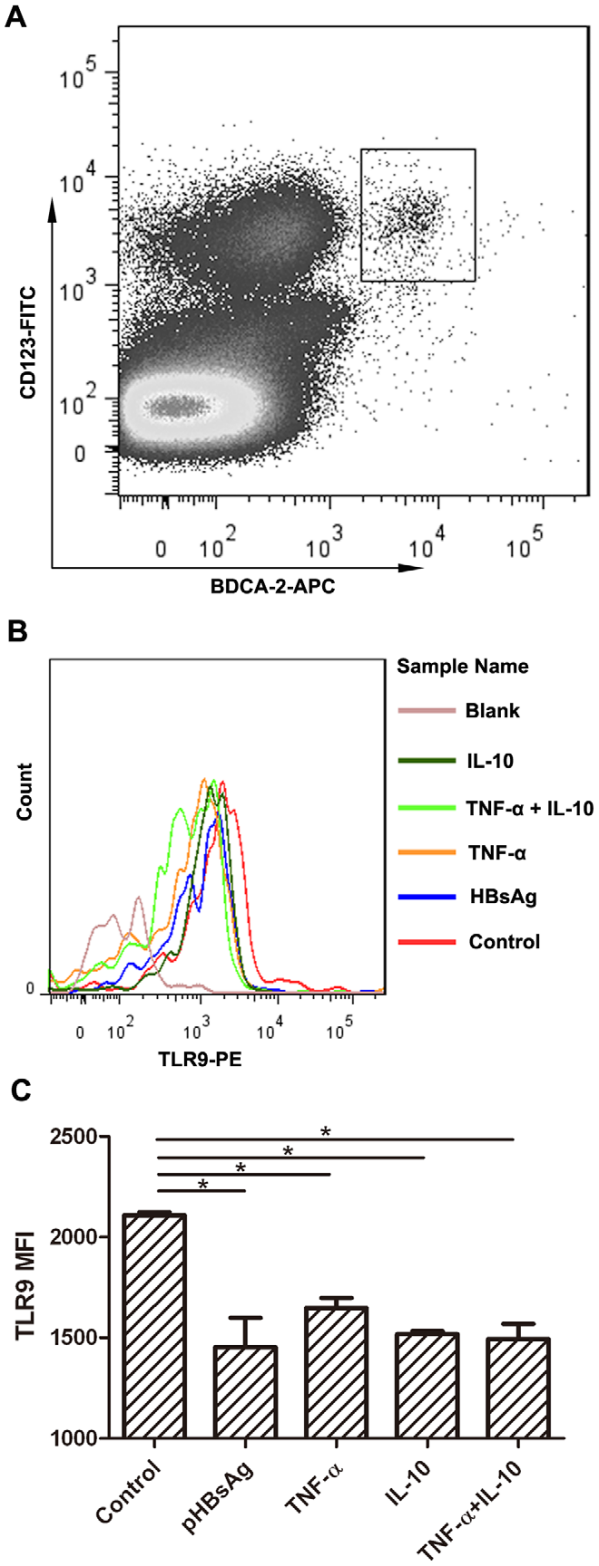
HBsAg downregulates the expression of TLR9 in pDCs. PBMCs were treated with 10 µg/ml pHBsAg and 10 ng/ml of exogenous TNF-α, IL-10 or TNF-α and IL-10 mixed together for 24 hours. The pDCs population was identified by BDCA-2-APC and CD123-FITC staining (A), and the TLR9 expression of pDCs was measured by intracellular staining. The representative MFI shift of the TLR9 expression in pDCs population from one donor was shown (B). And the statistical anslysis of TLR9 expression between different groups was performed using Student’s t test. (C).

Previous studies have indicated that TNF-α may modulate the expression of TLR9 on pDCs [20]. Therefore, we examined if TLR9 expression was changed by treatment with TNF-α or IL-10. PBMCs were treated with HBsAg (1 µg/ml), TNF-α (10 ng/ml) and IL-10 (10 ng/ml), and stained for flow cytometric analysis. The pDCs population was identified using surface markers BDCA-2 and CD123 (Fig. 6A). The MFI change of TLR9 expression in pDCs population was shown. Histogram representative of one assay showed the MFI shift indicating that HBsAg, TNF-α and IL-10 could moderately down-regulate the level of TLR9 expression as compared to the mock-treated control (Fig. 6B). Statistical analysis confirmed that HBsAg, TNF-α and IL-10 pretreatment on PBMCs could downregulate the TLR9 expression in pDCs (Fig. 6C), indicating a possible mechanism of decreased IFN-α producing ability in pDCs.

### 7. IL-10 Levels are Up-regulated in Chronic Hepatitis B Patients

**Figure 7:**
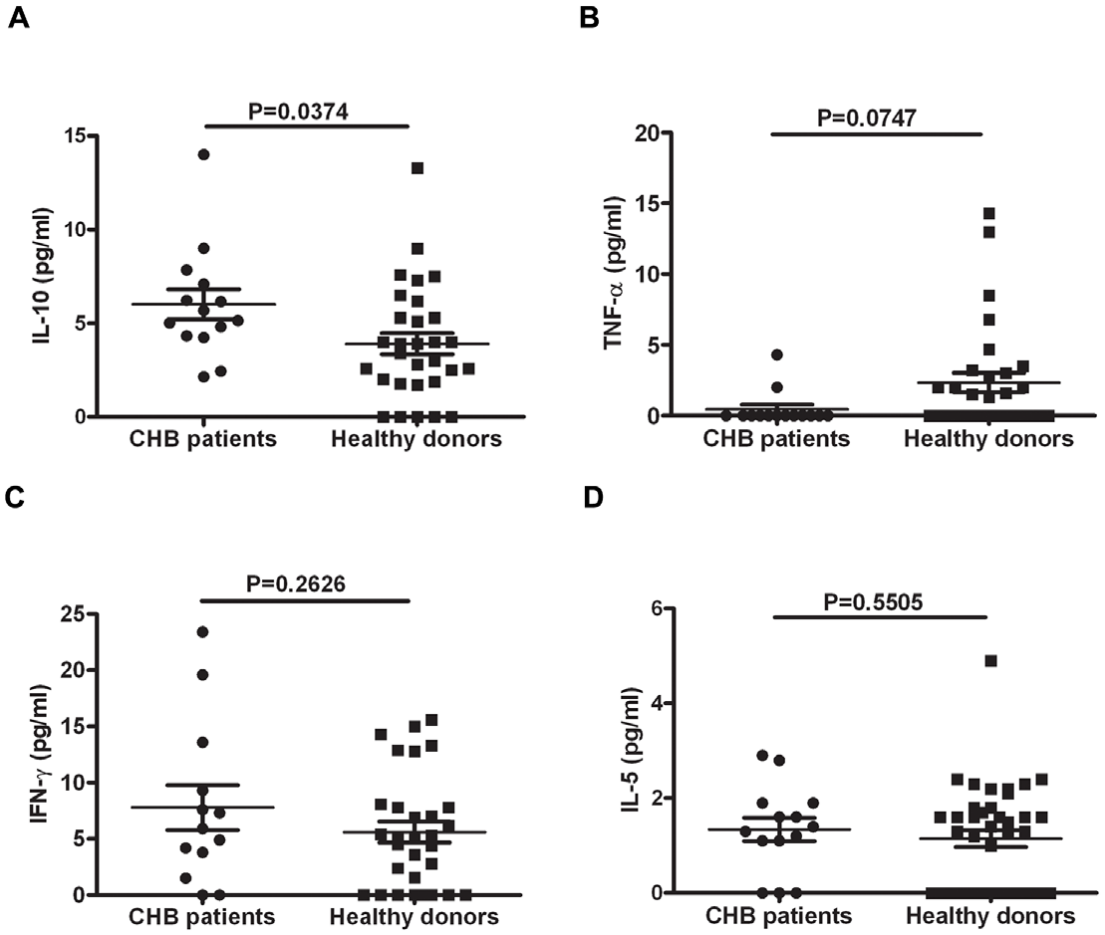
Serum IL-10 levels are elevated in CHB patients. The serum from 20 CHB patients and from 30 healthy donors was collected and cryopreserved at −80°C until analysis. For the serum cytokine quantification, a CBA assay was used, and IL-2, IL-4, IL-6, IL-10, TNF-α and IFN-γ levels were determined. Each dot represents one serum sample. The statistical analysis was performed using Student’s t test.

CHB patients have been reported to have decreased TLR9 expression [22]. Considering the importance of TNF-α and IL-10 in regulating the function of pDCs, we investigated whether serum TNF-α and IL-10 levels were up-regulated in CHB patients. The serum specimens obtained from healthy donors and CHB patients were analyzed by CBA. These results indicated that compared to the healthy donors, the serum levels of IL-10 were up-regulated in the CHB patients (Fig. 7A), in accordance with previous reports. The serum levels of TNF-α and other cytokines, such as IL-5 and IFN-γ, were not significantly different between the healthy donors and the CHB patients (Fig. 7B–7D). We also analyzed the correlation between the IL-10 levels and the serum HBsAg levels, but no obvious correlation was observed (data not shown). This lack of correlation may be a result of limited up-regulation of the IL-10 levels and a small patient cohort size.

## Discussion

Type I IFN is the first line of defense for the host against a viral infection because of its role in the induction of hundreds of ISGs that interfere with viral replication [23], [24]. Additionally, IFN-α initiates the host immune cascade to enable an effective host innate and adaptive immune response [25], [26]. Previous studies performed in transgenic mice have demonstrated that a deficiency in IFNAR1, PKR and IRF1 resulted in a significant increase in HBV replication [3]. These data indicated that IFN-α was an important cytokine for the control of HBV infection. pDCs are professional IFN-α-producing cells and play an important role in the immune response to viral infection. Previously, functional impairment of pDCs in CHB patients was reported by some research groups. In our previous study, we observed that HBsAg could inhibit the TLR9-related signaling pathway and decrease the production of IFN-α by pDCs [15]. To identify the mechanisms involved in this phenomenon, we continued to explore this phenomenon and determined that HBsAg acts on pDCs by inducing monocytes to secrete TNF-α and IL-10, which both mediate the inhibition of IFN-α production by pDCs. The relationship between HBsAg, monocytes and pDCs provides new insights into the ability of HBV to inhibit TLR9-mediated IFN-α production.

The mechanisms involved in the inhibition of pDC function by HBsAg and the HBV virion have been investigated by several groups. The direct targeting of either the TLR9 ligand-induced MyD88-IRF7-IFN-α signaling pathway or the mTOR-S6-IRF-7 signaling pathway induced by the HBV virion or HBsAg have been observed by our group, Vicente *et al.* and Woltman *et al.* [15], [16], [17]. In addition to studies by our group, Woltman *et al.* determined that HBV could inhibit pDCs both in a direct manner and that, in an indirect manner, monocytes in the peripheral blood were related with the indirect targeting of pDCs. In the current study, we have reported that HBsAg can indirectly inhibit the production of IFN-α by pDCs by modulating the signaling between monocytes and pDCs. This inhibition was observed to be mediated by TNF-α and IL-10 secretion. This suggests that there is a complicated network of immune signaling responses involved in the inhibition of pDCs by HBV.

The TNF-α and IL-10 that are secreted by monocytes mediated the inhibitory effect of HBsAg on pDCs. These data suggested that the two cytokines may play an important role in the viral escape from the host antiviral immune response. IL-10 typically provides balance in the host inflammatory immune response and negatively regulates IL-12 expression in Th1 differentiation. Various viruses, such as HCV and LCMV, have been observed to induce IL-10 production as a strategy to down-regulate the host immune response and allow their persistence in the host [27], [28]. The HBV core antigen can also stimulate IL-10 secretion by T cells and monocytes in the peripheral blood of CHB patients [29] and in PBMCs from healthy donors *in vitro* [17]. Here, we found that IL-10 induced by HBsAg could interfere with the production of IFN-α by pDCs. In contrast to IL-10, TNF-α is a pro-inflammatory cytokine. But similar to IL-10, TNF-α also clearly inhibited the secretion of IFN-α by pDCs. How these cytokines which have opposite inflammatory functions are coordinated in the regulation of the production of IFN-α by pDCs is still unknown and requires further study.

Our data also demonstrated that HBsAg could interfere with the expression of TLR9. These data are supported by a previous study by Xie *et al.* where TLR9 was observed to be down-regulated in the pDCs of CHB patients [22]. Recently, Vicent *et al.* determined that HBV virions could down-regulate the expression of both the TLR9 mRNA and protein in PBMCs by inhibiting the activity of TLR9 promoter. Whether the production of TNF-α or IL-10 can interfere with the TLR9 promoter activity and how the expression of TLR9 and its signal pathway is affected by HBV and HBsAg treatment requires further study.

During chronic HBV infection, large amounts of HBsAg are present in the blood of patients. HBsAg is thought to play a role in counteracting the host immune response to viral infection. Here, we report that HBsAg can impair the production of IFN-α by pDCs. These data are in accordance with a report by Woltman *et al.*, who observed that both HBV virion particle and HBsAg had an inhibitory effect on pDCs [15], [16]. However, Vincent’s group has reported that only the HBV virion particle, but not HBsAg, impaired the expression of TLR9 and the function of pDCs [17]. These conflicting data may be a result of the different origins of the HBsAg and the different concentration of HBsAg used in the experimental systems. The HBsAg we used was derived from the plasma of CHB patients. Western blot analysis indicated that the plasma-derived HBsAg was more glycosylated than the CHO-HBsAg or the yeast-expressed HBsAg, according to the 27 kD band of HBsAg identified (Fig. 2A and Fig. s1). Although the biological significance of the varying glycosylation levels of HBsAg is unclear, the glycosylation of the HBV structural protein may be an important mechanism for the virus to escape recognition by APCs. Evidence for this hypothesis is provided by Op den Brouw *et al.*, who indicated that branched oligosaccharide structures on HBV prevent its interaction with both DC-SIGN and L-SIGN, allowing it to subvert the host immune response [30]. In addition to different glycosylation levels, HBsAg derived from different origins are diverse in their phospholipid moieties. Vanlandschoot *et al.* has reported that recombinant HBsAg expressed by yeast can bind CD14 via LPS binding protein (LBP) catalysis and can therefore attach to the surface of monocytes. However, pHBsAg does not possess this property and cannot attach to monocytes [13]. Earlier studies have indicated that HBsAg concentrations in CHB patients could be as high as 100 µg/ml; however HBsAg levels in the majority of CHB patients in our study did not exceed 1 µg/ml. Therefore, we only used 1 µg/ml of pHBsAg, while Woltman *et al.* used 5 µg/ml of CHO-HBsAg in their experiments. Both groups observed a significant inhibition of IFN-α production, and it is hypothesized that an increase in HBsAg concentration should lead to higher levels of IFN-α inhibition. How the differences in the glycosylation and lipid content affect the function of HBsAg also requires further study.

Chronic HBV infection is a serious problem both in China and worldwide. The activation of pDCs leading to the production of IFN-α is important for an effective anti-HBV immune response. Our present study reported that HBsAg acted on pDCs in an indirect manner by inducing monocytes to secrete TNF-α and IL-10, which mediated the inhibition of IFN-α production by pDCs. With the new understanding of the relationship between HBsAg, monocytes, pDCs and the cytokine response, our study has provided a novel insight into the complicated mechanism by which HBV modulates the innate immune system. These data will help to further understanding how HBV establishes persistent infection and which may aid in the development of an effective antiviral treatment to the control of HBV infection.

## Supporting Information

Figure S1 Characterization of the serum-derived HBsAg (pHBsAg) and HBsAg produced in CHO cells (CHO-HBsAg) and HBsAg expressed by yeast (Yeast-HBsAg). 1 µg of pHBsAg, 0.76 µg of CHO-HBsAg and 1 µg Yeast-HBsAg were run on a 12% SDS-PAGE for subsequent silver staining and western blot analysis. The unglycosylated (24 kD) and glycosylated (27 kD) form of HBsAg were observed.
(TIF)
