# Supplementary figures and images for: HBsAg Inhibits IFN-α Production in Plasmacytoid Dendritic Cells through TNF-α and IL-10 Induction in Monocytes
Source: PLoS One. 2012 Sep 14;7(9):e44900. doi: 10.1371/journal.pone.0044900 (PMC3443230; doi:10.1371/journal.pone.0044900)

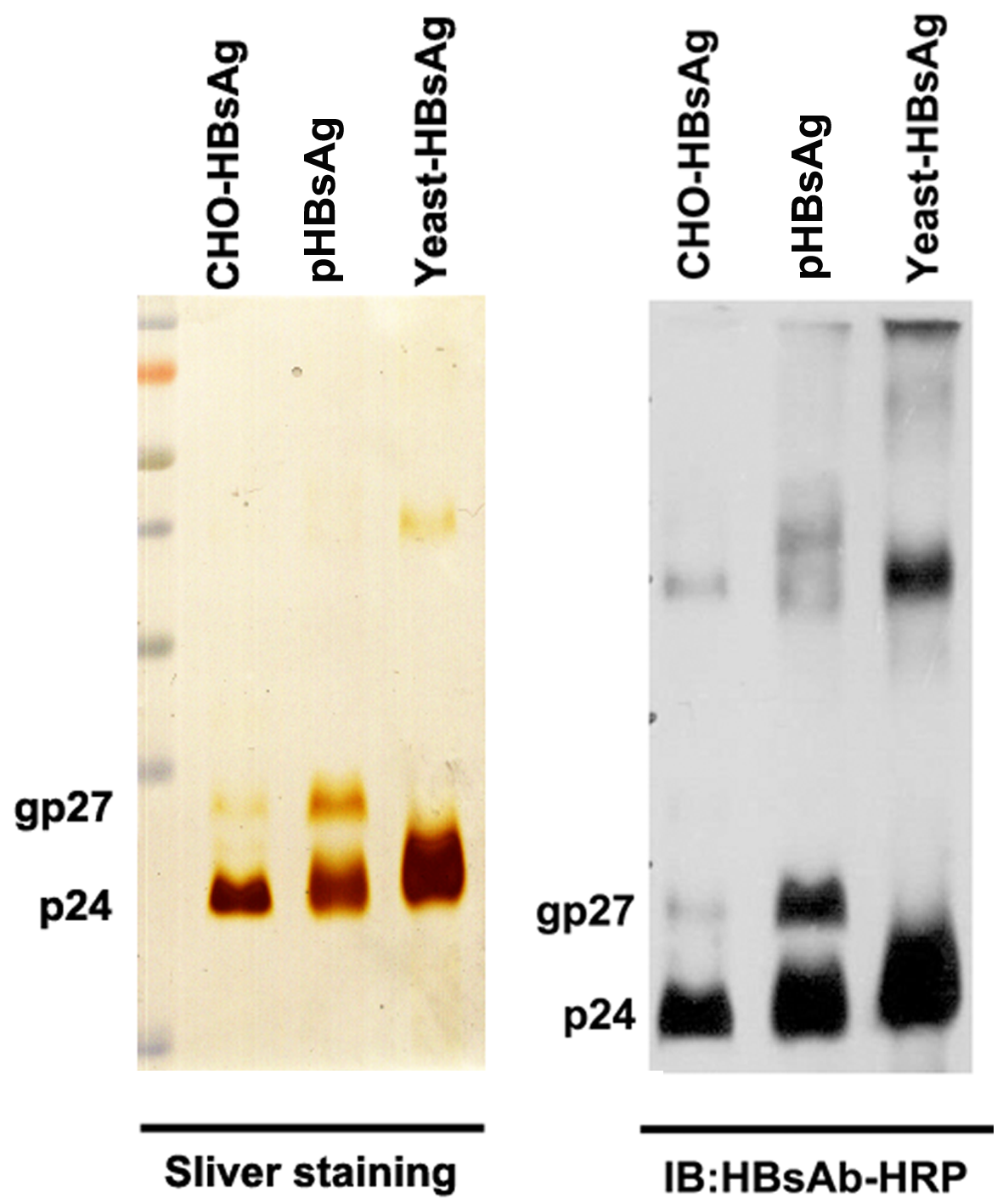

Supplement: Figure S1 — Characterization of the serum-derived HBsAg (pHBsAg) and HBsAg produced in CHO cells (CHO-HBsAg) and HBsAg expressed by yeast (Yeast-HBsAg). 1 µg of pHBsAg, 0.76 µg of CHO-HBsAg and 1 µg Yeast-HBsAg were run on a 12% SDS-PAGE for subsequent silver staining and western blot analysis. The unglycosylated (24 kD) and glycosylated (27 kD) form of HBsAg were observed. (TIF) [file pone.0044900.s001.tif]
